# Plant-Derived Antimicrobials Reduce *E. coli* O157:H7 Virulence Factors Critical for Colonization in Cattle Gastrointestinal Tract *In Vitro*

**DOI:** 10.1155/2014/212395

**Published:** 2014-06-19

**Authors:** Sangeetha Ananda Baskaran, Kumar Venkitanarayanan

**Affiliations:** ^1^Department of Poultry Science, Texas A&M University, College Station, TX 77843, USA; ^2^Department of Animal Science, University of Connecticut, Storrs, CT 06269, USA

## Abstract

This study investigated the effect of subinhibitory concentrations (SIC) of five plant-derived antimicrobials (PDAs), namely, trans cinnamaldehyde, eugenol, carvacrol, thymol, and *β*-resorcylic acid, on *E. coli* O157:H7 (EHEC) attachment and invasion of cultured bovine colonic (CO) and rectoanal junction (RAJ) epithelial cells. In addition, PDAs' effect on EHEC genes critical for colonization of cattle gastrointestinal tract (CGIT) was determined in bovine rumen fluid (RF) and intestinal contents (BICs). Primary bovine CO and RAJ epithelial cells were established and were separately inoculated with three EHEC strains with or without (control) SIC of each PDA. Following incubation, EHEC that attached and invaded the cells were determined. Furthermore, the expression of EHEC genes critical for colonization in cattle was investigated using real-time, quantitative polymerase chain reaction in RF and BICs. All the PDAs decreased EHEC invasion of CO and RAJ epithelial cells (*P* < 0.05). The PDAs also downregulated (*P* < 0.05) the expression of EHEC genes critical for colonization in CGIT. Results suggest that the PDAs could potentially be used to control EHEC colonization in cattle; however follow-up *in vivo* studies in cattle are warranted.

## 1. Introduction

Enterohemorrhagic* Escherichia coli* O157:H7 (EHEC) is a major food-borne pathogen that causes disease conditions in humans, ranging from diarrhea to hemorrhagic colitis and hemolytic-uremic syndrome [1]. Cattle are the principal reservoir of EHEC [2–5], with fecal contamination of carcass being an important source of human infection. In addition, fecal shedding of EHEC imposes a risk of direct zoonotic and environmental transmission of the pathogen to humans, especially children [6].

The primary site of EHEC colonization in cattle is the terminal rectum, particularly an anatomical area within the terminal rectum referred to as the rectoanal junction (RAJ) [3, 4]. The EHEC carriage at RAJ in cattle is associated with high levels of pathogen excretion in feces as well as long-duration of fecal shedding [3, 7–9]. Besides feces, rectal, colonic, and rumen contents were also found as sources of EHEC in cattle [4].

Previous research has revealed that EHEC colonization in cattle gastrointestinal tract (CGIT) is mediated by several factors. First, the bacterial colonization of CGIT is facilitated by its attachment to the gastrointestinal epithelial cells [10–12]. In addition, ethanolamine (EA) utilization, which is mediated by the induction of ethanolamine utilization genes (*eutB*,* eutC*, and* eutR*), is also critical for EHEC colonization [13]. Ethanolamine present in the CGIT could be selectively utilized by EHEC as an energy substrate. In addition, Hughes et al. [14] demonstrated the role of quorum sensing in EHEC colonization of CGIT. The pathogen produces SdiA protein, a LuxR homolog that senses acyl-homoserine lactones (AHLs) produced by endogenous microflora in the rumen. This SdiA-AHL chemical signaling regulates the expression of* gad* acid resistance system and locus of enterocyte effacement (LEE) genes in EHEC, which are essential for colonization in cattle [14]. Also, this signaling facilitates a commensal lifestyle in cattle gut. Furthermore, EHEC colonization is mediated by catabolism of complex oligosaccharides, namely, L-fucose, D-galactose, sialic acids,* N*-acetylgalactosamine, and* N-*acetylglucosamine, present in the intestinal mucus of cattle [15, 16]. Therefore, catabolism of rectal mucin-derived sugars, mainly* N*-acetylgalactosamine and L-fucose, plays a role in the colonization of EHEC in the bovine rectum. Hence, inhibiting SdiA-mediated colonization and minimizing EA and mucus carbohydrate utilization would aid in limiting EHEC colonization and shedding in cattle and, consequently, the food-borne transmission of EHEC.

The use of plant-derived antimicrobials (PDAs) for controlling pathogenic microorganisms has received increased attention in recent years due to concerns for toxicity of synthetic chemicals and emerging antimicrobial resistance in bacteria [17–20]. The PDAs represent a group of natural antimicrobials that have been traditionally used to preserve foods as well as enhance food flavor. The antimicrobial properties of several plant-derived essential oils have been demonstrated [21–23], and a variety of active components of these oils have been identified.* trans*-Cinnamaldehyde (TC) is an aromatic aldehyde present as a major component of bark extract of cinnamon (*Cinnamomum verum*) [22]. Eugenol (EG) is an active ingredient in the oil from cloves (*Eugenia caryophyllus*) [24]. Carvacrol (CR) and thymol (TH) are antimicrobial ingredients in oregano oil obtained from* Origanum glandulosum* [25], whereas *β*-resorcylic acid (BR; 2,4 dihydroxybenzoic acid) is a phytophenolic compound widely distributed among the angiosperms and is a secondary metabolite that plays a key role in the biochemistry and physiology of plants [26]. All the aforementioned compounds are classified as GRAS (generally recognized as safe) by the US Food and Drug Administration [26–28].

This study was undertaken to determine the effect of subinhibitory concentrations (SIC, the highest concentration below MIC that does not inhibit bacterial growth) of PDAs on EHEC invasion of cultured bovine colonic (CO) and rectoanal junction (RAJ) epithelial cells. In addition, the effect of PDAs on EHEC genes that are critical for colonization in CGIT was determined in bovine rumen fluid (RF) and intestinal contents (BICs) for potential future application as a dietary supplement for reducing EHEC carriage in cattle.

## 2. Materials and Methods

### 2.1. Bacterial Culture and Media

Three isolates of EHEC, namely, E10 (meat), E16 (meat), and E22 (calf feces), were used in this study. Each EHEC strain was individually cultured in 10 mL of tryptic soy broth (TSB, Becton-Dickinson, Sparks, MD) at 37°C for 24 h with agitation (150 rpm). Following incubation, the cultures were sedimented by centrifugation (3,600 ×g for 15 min), washed twice, and resuspended in 10 mL of sterile phosphate-buffered saline (PBS, pH 7.2). To determine the bacterial population in each culture, 0.1 mL portions of appropriately diluted culture were surface-plated on tryptic soy agar (TSA, Becton-Dickinson, Sparks, MD) and incubated at 37°C for 24 h. Appropriate dilutions of each isolate were made in PBS, and 0.1 mL (~6.0 log CFU) was used as the inoculum.

### 2.2. Determination of SIC of PDAs

The SIC of TC, EG, CR, TH, and BR against three EHEC strains was determined, as reported previously [28, 29]. Tryptic soy broth containing each of the aforementioned PDAs in the range of 0 to 0.05% (vol/vol) in increments of 0.001% was inoculated with each strain of EHEC at 6.0 log_10_ CFU/mL and incubated at 37°C for 24 h. Control samples containing TSB without any added PDAs were included. After 24 h of incubation, the samples were serially diluted (1 : 10) in PBS and bacterial counts were determined on TSA. The highest concentration of each plant compound that did not significantly reduce bacterial growth after incubation at 37°C for 24 h was taken as the SIC of the PDA.

### 2.3. Primary Cell Cultures of Bovine Colon and Rectal-Anal Junction Epithelial Cells

The bovine colonocytes and RAJ epithelial cells were isolated, as previously described [30, 31]. Fresh bovine colonic and RAJ tissues were obtained from a local slaughterhouse. The tissues were immediately transferred to the laboratory in cold isotonic NaCl solution supplemented with 100 U/mL penicillin, 100 *μ*g/mL streptomycin, 2.5 *μ*g/mL gentamicin, and 2.5 *μ*g/mL amphotericin. The epithelium was scraped from the underlying tissue and triturated in Hank's balanced salt solution (HBSS). The cell suspension was centrifuged at 130 g for 5 min and the pellet was resuspended in cold HBSS. The washing was repeated three times. The final pellet was resuspended in “dissociation solution” containing 10 mL HBSS, 10 mL Dulbecco's modified Eagle's medium (DMEM), 100 U/mL penicillin, 100 *μ*g/mL streptomycin, 2.5 *μ*g/mL gentamicin, 2.5 *μ*g/mL amphotericin, and 100 U/mL collagenase *Ι*. The cells were incubated with shaking for 45 min at 37°C. After digestion, the cell material was centrifuged through a 2% sorbitol gradient in DMEM at 50 g for 5 min, and the pellet was resuspended in 2% sorbitol gradient. This procedure was repeated until the supernatant was clear, and the resulting pellet was resuspended in an aliquot of DMEM. The identity of bovine colon and RAJ epithelial cells was confirmed by detecting the basal level expression of pan-cytokeratin gene (KRT7) [32] and absence of expression of vimentin gene (VIM, [32]) to rule out contamination of fibroblasts, by RT-qPCR.

### 2.4. Culture Medium

The culture medium used for growing cattle colonocytes and RAJ epithelial cells was DMEM supplemented with 100 U/mL penicillin, 100 *μ*g/mL streptomycin, 2.5 *μ*g/mL gentamicin, 2.5 *μ*g/mL amphotericin, 2.5% fetal bovine serum (FBS; Gibco Invitrogen), 30 ng/mL epidermal growth factor (EGF; Sigma-Aldrich), and 0.25 U/mL insulin (Sigma-Aldrich) [32].

### 2.5. Effect of PDAs on EHEC Adherence to Bovine Colon and RAJ Epithelial Cells

*E. coli* O157:H7 adherence to bovine colonic and RAJ epithelial cells was assayed, as described by Sheng et al. [32], with slight modifications. The cells were cultivated at 37°C under 5% CO_2_ atmosphere in a cell culture flask. Polystyrene (24-well) plates were seeded with cells at a density of 1 × 10^5^ cells per well and allowed to form a monolayer. The cell monolayer was washed three times with DMEM and each EHEC strain (6 log CFU/mL) was separately added and treated with the respective SIC of TC, EG, CR, TH, or BR. The cells were incubated for 2 h and washed three times with PBS. The cells were then lysed by treating with 0.1% triton X-100 for 15 min, and adherent EHEC population was quantified by plating on TSA. Each treatment was assayed in duplicate and the entire experiment was repeated three times.

### 2.6. Effect of PDAs on EHEC Invasion of Bovine Colon and RAJ Epithelial Cells

The invasion assay was performed as described previously by Sheng et al. [32]. The cells were cultivated at 37°C under 5% CO_2_ atmosphere in a cell culture flask. Polystyrene plates were seeded with cells at a density of 1 × 10^5^ cells per well and allowed to form a monolayer. The monolayer was washed three times with DMEM and each EHEC inoculum containing 6 log CFU/mL was added and treated with the SIC of each PDA. The cells were incubated for 2 h and washed three times with PBS. The cell monolayer was treated with 100 *μ*g of gentamicin per mL in culture medium for 2 h to kill all extracellular bacteria. Intracellular EHEC was enumerated after lysis with 0.1% triton X-100 and plating on TSA.

### 2.7. Effect of PDAs on EHEC Ability to Survive in Minimal Medium Containing EA

The ability of EHEC to survive/grow by using EA as sole nitrogen source was studied in M9 minimal medium [13] supplemented with EA hydrochloride (30 mM) and SIC of TC, EG, CR, TH, or BR. The minimal medium was inoculated with ~4 log CFU/mL of each EHEC strain, and bacterial counts were determined at 24 h of incubation at 37°C.

### 2.8. Bovine Rumen Fluid and Intestinal Contents

Rumen fluid (RF) was collected fromcattle that were housed at the University of Connecticut beef barn. Bovine intestinal contents (BICs) were collected from a local slaughterhouse. Both RF and BIC were filtered through a cheese cloth and centrifuged at 10,000 g for 15 min [13]. The supernatant obtained after centrifugation was filter-sterilized through 0.2 *μ*m filter. Sterile RF and BICs obtained were used for the experiment.

### 2.9. Effect of PDAs on EHEC Colonization Genes

The effect of TC, EG, CR, TH, and BR at their respective SICs on EHEC genes, namely,* gadA*,* gadC*,* gadX*,* ler*,* sdiA*,* eae*,* eutB*,* eutC*,* eutR*,* agaA*,* fucA*, and* fucO*, that are essential for its colonization in CGIT was investigated in RF and BICs as* in vitro* models.

#### 2.9.1. RNA Isolation

Sterile RF and BICs with or without SICs of the PDAs were inoculated with each EHEC strain (6.0 log CFU/mL) separately and incubated at 39°C for 4 h in an anaerobic chamber. Total RNA was extracted using RNeasy mini kit (Qiagen).

#### 2.9.2. cDNA Synthesis and Real-Time Quantitative PCR (RT-qPCR)

The RNA was converted to cDNA using the Superscript II reverse transcriptase kit (Invitrogen, Grand Island, NY). The cDNA was used as the template for real-time PCR amplification of the abovementioned cattle colonization genes. Primers specific for each of the aforementioned genes were designed from published GenBank sequences using Primer Express software (Applied Biosystems, Foster City, CA) (Table 1). Relative gene expression of the aforementioned genes was determined using a 7500 fast real-time PCR system (Applied Biosystems) with SYBR Green reagents. Data were normalized to the endogenous control, 16S rRNA, and the level of gene expression between treated and control samples was analyzed using the comparative threshold cycle method (C_T_). Each treatment had two samples and the experiment was replicated two times.

### 2.10. Statistical Analysis

For each treatment, data from independent replicate experiments were pooled and analyzed using PROC MIXED of SAS version (SAS Institute, Cary, NC, U.S.A.). Variation among replicates was used as the error term. Data were expressed as least squares means, and differences were considered significant at *P* < 0.05. Data comparisons for the gene expression study were made by using Student's *t*-test. Differences were considered significant when the *P* value was less than 0.05.

## 3. Results

**Figure 1:**
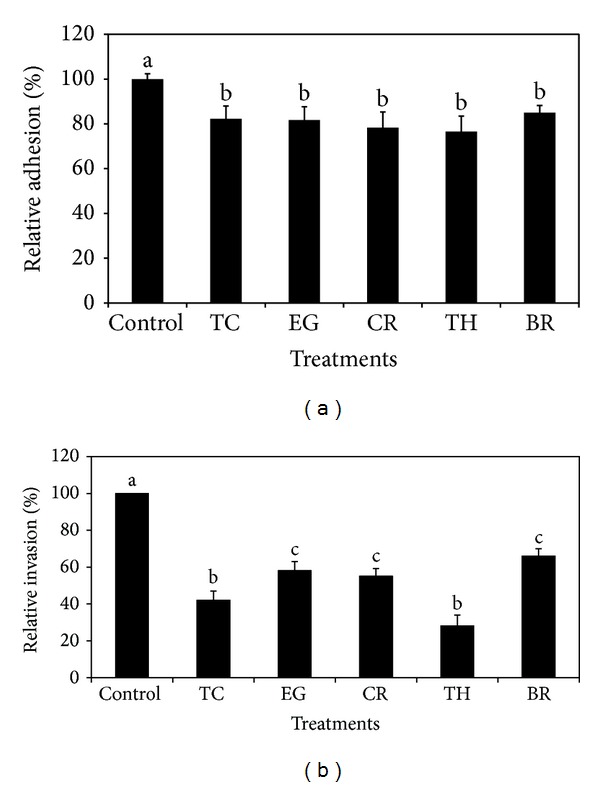
(a) Effect of PDAs on EHEC adhesion of bovine colonic epithelial cells. Treatments include control (0 mM), TC (0.75 mM), EG (1.85 mM), CR (0.65 mM), TH (0.65 mM), and BR (2.6 mM). Error bars represent SEM (*n* = 3). Bars with a common letter are not significantly different (*P* > 0.05). (b) Effect of PDAs on EHEC invasion of bovine colonic epithelial cells. Treatments include control (0 mM), TC (0.75 mM), EG (1.85 mM), CR (0.65 mM), TH (0.65 mM), and BR (2.6 mM). Error bars represent SEM (*n* = 3). Bars with a common letter are not significantly different (*P* > 0.05).

**Figure 2:**
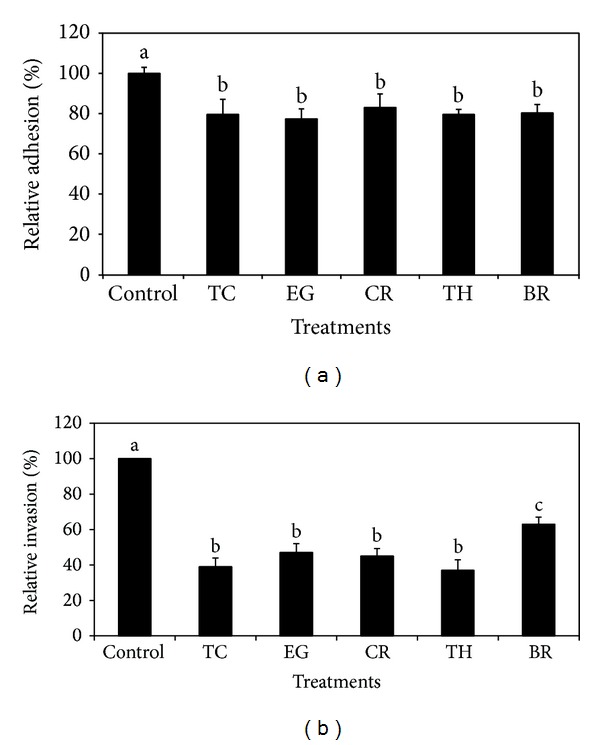
(a) Effect of PDAs on EHEC adhesion to bovine RAJ epithelial cells. Treatments include control (0 mM), TC (0.75 mM), EG (1.85 mM), CR (0.65 mM), TH (0.65 mM), and BR (2.6 mM). Error bars represent SEM (*n* = 3). Bars with a common letter are not significantly different (*P* > 0.05). (b) Effect of PDAs on EHEC invasion of bovine RAJ epithelial cells. Treatments include control (0 mM), TC (0.75 mM), EG (1.85 mM), CR (0.65 mM), TH (0.65 mM), and BR (2.6 mM). Error bars represent SEM (*n* = 3). Bars with a common letter are not significantly different (*P* > 0.05).

The RT-qPCR results revealed that the colon and RAJ epithelial cells constitutively expressed the pan-cytokeratin gene KRT7, thereby validating their identity (data not shown). In addition, the VIM gene encoding vimentin was not detected in both cell types, thus confirming that the isolated primary epithelial cells were devoid of any fibroblast contamination. Although the effect of PDAs on EHEC adhesion and invasion of colon and RAJ epithelial cells was investigated on three different strains of the pathogen, only results obtained with E16 strain are presented, since the effect of PDAs was not significantly different among the three EHEC strains (*P* > 0.05). The SIC of TC, EG, and BR was 0.75 mM (0.01%), 1.85 mM (0.03%), and 2.60 mM (0.04%), respectively, whereas that of CR and TH was 0.65 mM (0.01%). The SIC of PDAs obtained in TSB was similar in RF and BICs. The effect PDAs on EHEC adhesion and invasion of bovine colonocytes is provided in Figures 1(a) and 1(b). In the adhesion assay, all the PDAs reduced EHEC adhesion by ~20% (*P* < 0.05) compared to control (Figure 1(a)). The PDAs were also effective in inhibiting the invasive ability of EHEC to colonocytes by 40–80% (*P* < 0.05). Thymol was found to be more effective in reducing EHEC invasion of colonocytes (*P* < 0.05). The effect of PDAs on EHEC adhesion and invasion of RAJ epithelial cells is depicted in Figures 2(a) and 2(b). As observed with the colonocytes, PDAs significantly reduced adhesion and invasion of EHEC to RAJ cells by ~20% and 60%, respectively (Figure 2(a)).

**Figure 3:**
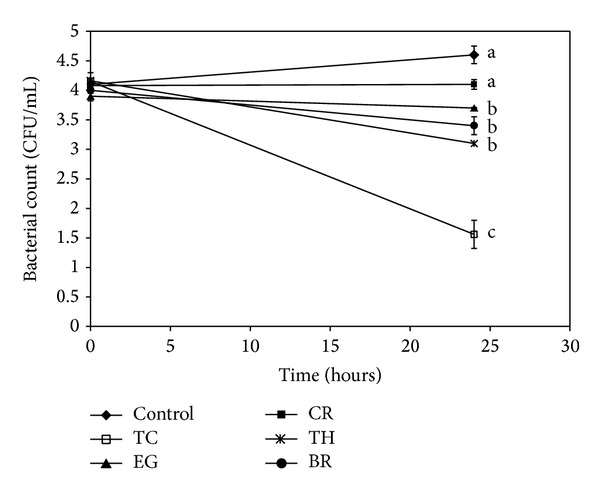
Effect of PDAs on EHEC ability to utilize ethanolamine in minimal medium. Treatments include control (0 mM), TC (0.75 mM), EG (1.85 mM), CR (0.65 mM), TH (0.65 mM), and BR (2.6 mM). Error bars represent SEM (*n* = 3). Lines with a common letter are not significantly different (*P* > 0.05).

**Figure 4:**
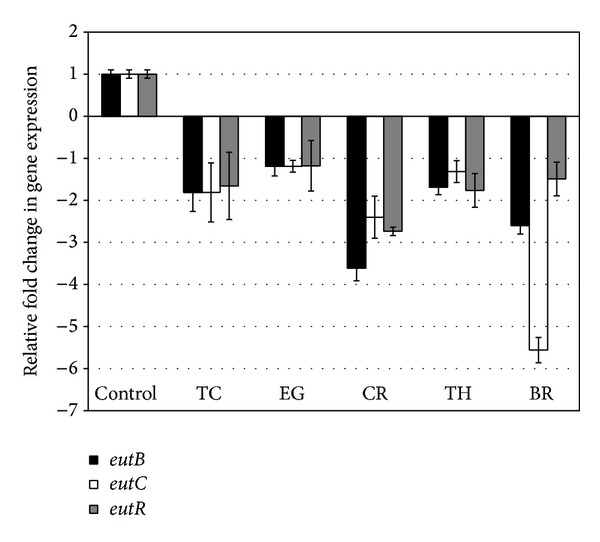
Effect of PDAs on EHEC ethanolamine utilization genes in RF. Treatments include control (0 mM), TC (0.75 mM), EG (1.85 mM), CR (0.65 mM), TH (0.65 mM), and BR (2.6 mM). Error bars represent SEM (*n* = 3). *All the treatments are significantly different from control at *P* < 0.05.

**Figure 5:**
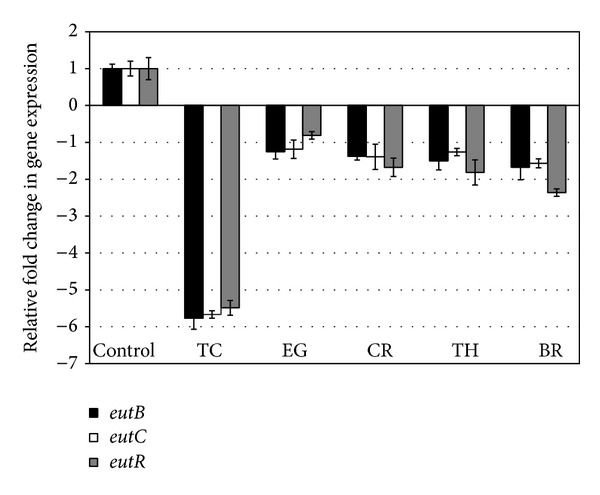
Effect of PDAs on EHEC ethanolamine utilization genes in BICs. Treatments include control (0 mM), TC (0.75 mM), EG (1.85 mM), CR (0.65 mM), TH (0.65 mM), and BR (2.6 mM). Error bars represent SEM (*n* = 3). *All the treatments are significantly different from control at *P* < 0.05.

**Figure 6:**
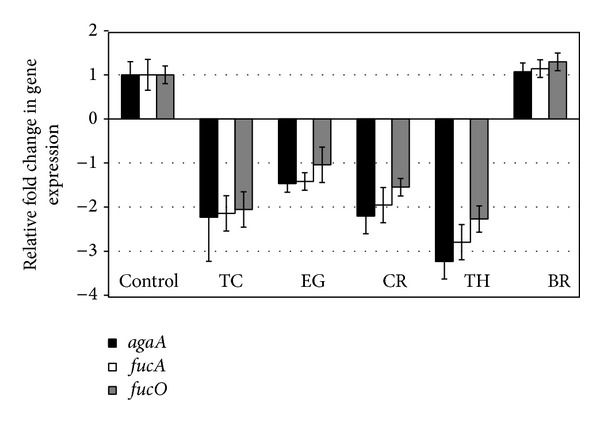
Effect of PDAs on EHEC mucus utilization genes in RF. Treatments include control (0 mM), TC (0.75 mM), EG (1.85 mM), CR (0.65 mM), TH (0.65 mM), and BR (2.6 mM). Error bars represent SEM (*n* = 3). *All the treatments except BR are significantly different from control at *P* < 0.05.

**Figure 7:**
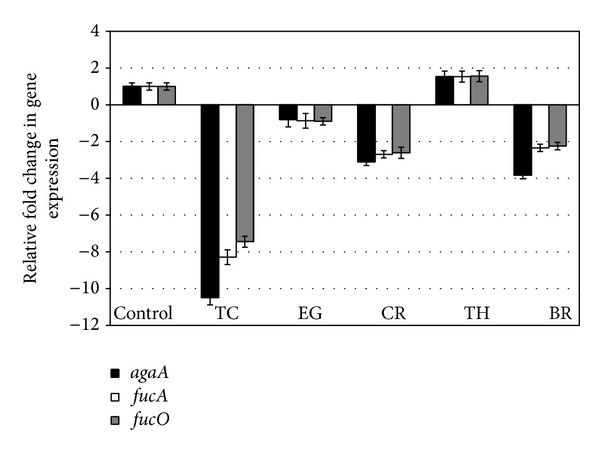
Effect of PDAs on EHEC mucus utilization genes in BICs. Treatments include control (0 mM), TC (0.75 mM), EG (1.85 mM), CR (0.65 mM), TH (0.65 mM), and BR (2.6 mM). Error bars represent SEM (*n* = 3). *All the treatments except TH are significantly different from control at *P* < 0.05.

**Figure 8:**
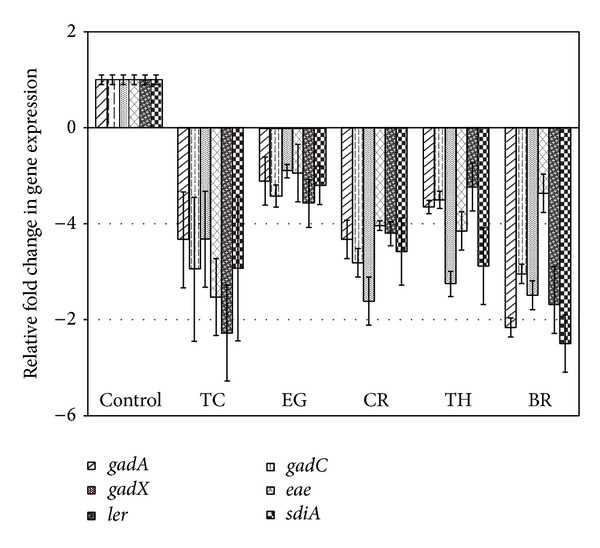
Effect of PDAs on EHEC SdiA-mediated colonization genes in RF. Treatments include control (0 mM), TC (0.75 mM), EG (1.85 mM), CR (0.65 mM), TH (0.65 mM), and BR (2.6 mM). Error bars represent SEM (*n* = 3). *All the treatments are significantly different from control at *P* < 0.05.

**Figure 9:**
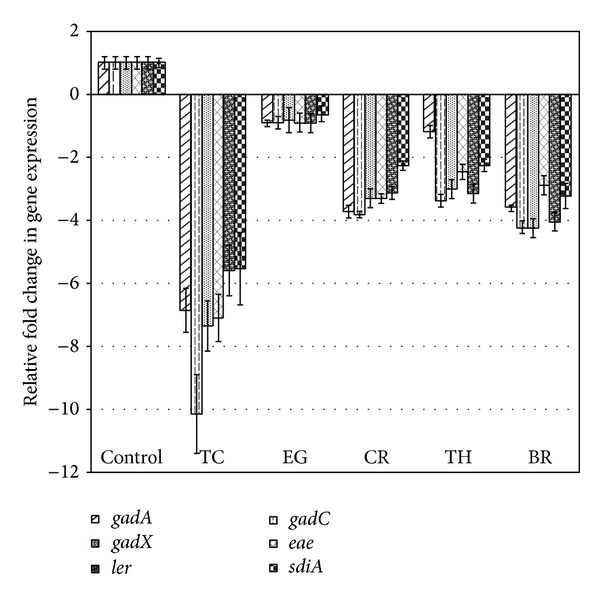
Effect of PDAs on EHEC SdiA-mediated colonization genes in BICs. Treatments include control (0 mM), TC (0.75 mM), EG (1.85 mM), CR (0.65 mM), TH (0.65 mM), and BR (2.6 mM). Error bars represent SEM (*n* = 3). *All the treatments are significantly different from control at *P* < 0.05.

The effect of PDAs on EHEC ability to survive/grow by utilizing EA as a nitrogen source is shown in Figure 3. The control and all PDA treatments had a bacterial population of ~4 log CFU/mL at the start of the assay. After 24 h, EHEC grew by 0.5 log CFU/mL in control samples devoid of any PDA, whereas the bacterial count in samples containing PDAs except EG decreased significantly (*P* < 0.05). Among the various PDAs tested, TC was most effective in reducing EHEC's ability to survive, where the pathogen counts were reduced to ~1.5 log CFU/mL. To elucidate the mechanism of action of PDAs on EHEC ability to utilize EA as energy source, an RT-qPCR was performed on RNA obtained from EHEC grown in RF and BICs, using primers specific for ethanolamine utilization genes, namely,* eutB*,* eutC*, and* eutR*. The results are given in Figures 4-5. The results revealed that all the PDAs significantly downregulated (*P* < 0.05) the expression of the three EA utilization genes.* beta*-Resorcylic acid was most effective in reducing* eutC* expression in RF (Figure 5), whereas TC brought about maximal reductions in all three EA utilization genes in BICs, which concurred with the findings from the survival assay (Figure 3). Similarly, RT-qPCR data indicated that the PDAs, except BR in RF and EG and TH in BICs, downregulated* agaA*,* fucA*, and* fucO* involved in EHEC mucin utilization in cattle (*P* < 0.05) (Figures 6 and 7). Likewise, the expression of SdiA-controlled EHEC colonization genes was also decreased by the PDAs except EG (*P* < 0.05) (Figures 8 and 9).

## 4. Discussion

Since cattle serve as the principal reservoir of EHEC, decreasing the carriage of* E. coli* O157:H7 in the cattle gastrointestinal tract would potentially lead to decreased fecal shedding, which in turn would improve farm and animal hygiene and reduce contamination of food products such as beef, raw milk, and fresh produce [33–35]. Thus, there is a critical need for effective preharvest interventional approaches to decrease* E. coli* O157:H7 carriage and shedding by cattle [36].

In ruminants, rumen microorganisms utilize feeds to produce volatile fatty acids and protein as an energy and protein supply for the animals, respectively [37]. This fermentation process, however, potentially results in energy and protein inefficiencies by loss of methane and ammonia, respectively [38], thereby leading to reduced performance of the animal and release of methane into the environment [39]. In cattle, therefore, supplementation of antibiotic ionophores in feed has been reported as a useful strategy for reducing energy and nitrogen losses in the rumen [40]. However, the use of antibiotics in feeds has been prohibited in many countries due to potential residues in foods and the emergence of antibiotic resistant bacteria. This has led to exploring alternative approaches to antibiotics, including supplementing antimicrobial plant extracts, for modulating rumen fermentation [37, 41]. This study investigated the potential of several plant-derived antimicrobials for attenuating EHEC virulence factors that are critical for colonization in CGIT. Since subinhibitory concentrations of antimicrobials, including antibiotics, can modulate bacterial physicochemical functions, including that of genes, they are used for studying the effect of antimicrobials on bacterial gene expression and virulence [42–44]. Therefore, we investigated the potential inhibitory effect of SIC of TC, EG, CR, TH, and BR on EHEC virulence factors critical for intestinal colonization in cattle. Moreover, since the intended application of the PDAs is as dietary supplements in cattle to control EHEC colonization, lowest effective concentrations of the plant molecules are advantageous for economical and palatability reasons.

It is demonstrated that EHEC primarily colonizes the terminal rectum, especially the rectoanal junction in bovines, besides colon [3, 4]. In addition, Sheng et al. [32] demonstrated that EHEC was internalized by RAJ epithelial cells, which plays a major role in the persistence of this bacterium in cattle. Hence, we first investigated the effect of PDAs on adherence and invasion of EHEC in bovine colonic and RAJ epithelial cells. To accomplish this, primary cells from bovine colonocytes and RAJ epithelial cells were isolated and their identity was confirmed by detecting markers specific to the epithelial cells. The results from the cell culture studies revealed that all the PDAs significantly reduced the adhesive and invasive abilities of EHEC to both primary epithelial cell lines (Figures 1(a) and 1(b) and Figures 2(a) and 2(b)). The reduced adhesive and invasive abilities of EHEC observed in the cell culture studies were supported by RT-qPCR data, where the results revealed that the PDAs significantly downregulated the expression of* eae* and* ler* (*P* < 0.05), which play a critical role in bacterial colonization in CGIT [14, 45, 46].

In addition, we determined the effect of the PDAs on EHEC ability to utilize ethanolamine for survival, since Bertin et al. [13] demonstrated the presence of ethanolamine in the gastrointestinal tract of cattle and EHEC ability to metabolize it for energy. This gives a competitive edge to EHEC to utilize EA as a source of nitrogen, which is not used by resident microbiota, since they lack the genes for EA utilization, thus favoring EHEC persistence in the bovine intestine [13]. Hence, gastrointestinal contents, namely, RF and BICs, were used as* in vitro* models for determining the effect of PDAs on EHEC's ability to persist by utilizing EA. The results revealed that TC, CR, TH, and BR significantly reduced the pathogen's ability to persist when EA was the primary nitrogen source in the growth medium (Figure 3). These results concurred with the gene expression studies, where a significant downregulation in majority of the EA utilization genes was brought about by the PDAs (Figures 4 and 5).

The mucins present in the intestinal mucus of cattle consist of proteins extensively glycosylated by* O*-linked oligosaccharides [15]. The intestinal mucins provide a substrate for energy and can be utilized by EHEC for its growth. Snider et al. [16] showed that two major sugars present in mucin, namely, N-acetyl-galactosamine and fucose, play a critical role in EHEC colonization in cattle. Hence, we studied the PDAs' effect on mucus carbohydrate utilization genes in EHEC. Among the tested PDAs, TC was found to be consistently effective in decreasing the expression of* agaA*,* fucA*, and* fucO* both in RF and BICs (*P* < 0.05).

Besides EA utilization, SdiA-mediated chemical sensing plays a major role in EHEC colonization in the bovine gastrointestinal tract [14, 47]. Hughes et al. [14] demonstrated the importance of SdiA-mediated chemical sensing in reducing EHEC colonization in the bovine gastrointestinal tract. These investigators found that an* sdiA* mutant of EHEC was defective for colonization in CGIT and concluded that interventions targeting SdiA in EHEC are a potential strategy to control the pathogen in cattle. Our results show that the PDAs significantly reduced the expression of not only sdiA, but also several other SdiA-controlled genes critical for colonization in cattle, includingthe* gad* acid resistance system,* eae,* and* ler* (Figures 8 and 9).

In conclusion, the results from this study suggest the potential efficacy of one or more of the PDAs, especially TC, as a dietary supplement for reducing EHEC shedding in cattle, but extensive follow-up studies in cattle are needed for validating their use.

## Figures and Tables

**Table 1:** List of primers used in this study.

Gene	Forward primer sequence (5′-3′)	Reverse primer sequence (5′-3′)
*sdiA *	TGGCGGACGGGTGAGTA	CCGGAGTTATCCCCAACTTACA
*eae *	GTTGTTGCCGGCGTTACAC	CGCGATAATTGCTTTGAAAAGA
*ler *	GCGGTCAACCGTTCCA	TGAGGCTCGTGAGGAATACGA
*gadA *	TCGGACCATTGTAGTCATCTTGA	CACAAATTCGGCATGCAGTT
*gadC *	CGCAGCTCCGCATGATATT	GATTATCCGCGGACCAACTAAG
*gadX *	TCTCCGCCTGCAAGTCCTA	TCGATTTCATCCGCGTGTT
*eutB *	GCGTGGATCCGCATGAAT	GCATCCGCAGCACTTTGAAT
*eutC *	CGTCGTCATCCAGGATTGC	TGCTATGGCTTTCCTTCTTTTTTT
*eutR *	CTACAGCTGGGATTGCGGTAA	TGCTTGCGGATGCGATT
*agaA *	AATGTAACAGACACGGTCTCACAAA	TCCCTAATCTATCCGCCTGAAG
*fucA *	CGAAAGTACAAGCGGAGACTATCA	GTTTCTGCAAAAGCATCATCTGA
*fucO *	AAAGCAGCTGAAACAACTAATGGA	CACGCGCAACTTCGGTATT
